# Electrical production data of a domestic grid-connected rooftop PV plant in normal and shading faults conditions associated with solar and meteorological data in a tropical climate

**DOI:** 10.1016/j.dib.2022.108723

**Published:** 2022-12-19

**Authors:** Alexandre Graillet, Carole Lebreton, Chao Tang, Fabrice Kbidi, Tifenn Jegado, Cédric Damour, Michel Benne

**Affiliations:** ENERGY-Lab, Université de La Réunion, 15, Avenue René Cassin CS 92003, CEDEX 9, Saint-Denis 97744, France

**Keywords:** Photovoltaic system, Shading faults, Electrical production, Temperature, Solar irradiance, NoSQL database

## Abstract

The proposed measured data combines PV plant electrical data with associated solar and meteorological data during normal and faulty conditions. Data are collected regarding a domestic rooftop PV plant of 4 kW, located in the La Réunion Island, in the South-West of Indian Ocean. The present dataset includes healthy behavior and different types of shading faults, identified and labelled by means of a numeric variable. The electrical data (voltage, current and power at AC and DC side as well as produced energy and grid frequency) are collected thanks to PV inverters. Global and diffuse irradiance, PV temperature and ambient temperature are acquired thanks to additional sensors. Electrical and meteorological data sampling frequencies are set to 0.2 Hz and 1 Hz respectively. At present, 12 months of data are available and the database is still being updated. The data streams from each connected device require proper techniques to ensure their persistence. To be able to provide both efficient ingestion and retrieval of these time series collections, the NoSQL database management system InfluxDB has been implemented. The whole dataset is available on Zenodo repository, and can be used, for instance, for PV modeling, PV plant behavior analysis, PV production forecasting and PV Fault Detection and Diagnosis (FDD) tool development.

**Specifications Table**

**Table:** 

Subject	Renewable Energy, Sustainability and the Environment
Specific subject area	PV system modeling, fault detection and diagnosis.
Type of data	Tables
How the data were acquired	The data was measured directly on the PV installation. The electrical data (voltage, current and power at AC and DC side as well as produced energy and grid frequency) are collected via the PV system's own Energrid module. Solar irradiance data was measured by a Delta-T Device SPN1 in the vicinity of the solar panels and at the same tilt. Ambient and PV module temperature data were measured with two thermocouples, T-type and K-type respectively.
Data format	Raw
Description of data collection	Solar and temperature instruments are connected to a Campbell Scientific CR1000X datalogger for data collection. Data transmission is ensured via TCP/IP. Instantaneous measurements are made for each variable every second. The electrical data comes from the Energrid data logger developed to monitor grid-connected PV systems. The frequency of the measurements is defined using Gridsoft software for the operation and management of grid-connected photovoltaic systems equipped with Energrid.
Data source location	Data are measured on a rooftop PV plant, installed on a Faculty of Science building at the University of La Réunion. Energy Lab, University of La Réunion Saint-Denis, La Réunion, Indian Ocean France −20.90126, 55.48422.
Data accessibility	Repository name: Zenodo Data identification number: 10.5281/zenodo.7157424 Direct URL to data: 10.5281/zenodo.7157424
Related research article	[1] Lebreton C, Kbidi F, Graillet A, Jegado T, Alicalapa F, Benne M, Damour C. PV System Failures Diagnosis Based on Multiscale Dispersion Entropy. *Entropy*. 24 (2022) 1311. 10.3390/e24091311

## Value of the Data

• PV plant electrical data, combined with irradiance and temperature data, are valuable data in the context of renewable energy generalization. Tropical climate induces worsened aging and increases the difficulties of PV modeling. The high sampling (1 Hz) rate allows to follow up the production as well as irradiance and temperature variations with high accuracy.
• Shading faults are common on PV plants, generally caused by vegetation, buildings around or soiling that provoke drop in performance and premature degradation of the modules. This shading faults implementation on this installation thus provides valuable insights on PV behavior and its electrical production.
• PV electrical data (Power, voltage and current at DC and AC sides) complemented by solar and meteorological data (global and diffuse irradiance, ambient and PV cell temperatures), can be used to build PV system models (Ayop et al. [2]), as close as possible to real aging and faulty PV plants. These data are useful for PV plant production forecasters, for considering shading issues, and for Researchers working on PV Fault Detection and Diagnosis (FDD) as Rouani et al. [3].
• Effects of shading faults on PV plants can be observed in detail and analyzed as Bansal et al. [4]. The indication of the type of shading fault (Partial Shading, Uniform Shading …) is directly filled in the database as numeric variable. This carefully labelled indication can be very useful to conduct Machine Learning tools, as supervised classification.
• The solar radiation measurements could be used to recalibrate other grided dataset, e.g., meteorological satellite images, to obtain a better spatial coverage. The solar radiation measurement is of crucial importance for quantifying the variability / intermittency of solar resources.
• The solar radiation data combined with the production data is useful for micro-grid simulation and Energy Management System (EMS) development, in order to improving the optimization algorithms that constitute it. From the recorded measurements, it is also possible to deduce the solar production of a site in kWh to obtain, for example, the dimensioning of photovoltaic plants.

## 1 Objective

The data generation takes place in the experimental part of DETECT (Diagnosis onlinE of sTate of hEalth on eleCTrical systems) project at the University of La Réunion. DETECT is a research project aiming to develop a low-cost and low-tech fault diagnosis tool for electrical power systems, specifically PV systems undergoing tropical and humid climates. In the objective to add a minimum of external devices, only already available data on a common PV plant have been collected (data available via PV inverters, and radiometric station) during healthy and faulty behavior. The data are used to test the investigated DETECT fault diagnosis tool. The present data have already been partially used in this way and the results have been published [1].

## 2 Data Description

Three datasets are presented below:

• Dataset 1: “dt1_solar_and_meteorological_measurement.csv” corresponds to the solar and meteorological measurements from the sensors positioned on the photovoltaic installations (**cf.** Fig. 2). Variables of the first dataset are described in Table 1. Data are stored by a data logger with a sampling frequency of 1 Hz starting from 2021 to 10–28 for a minimum period of 12 months (YYYY-MM-DD hh:mm:ss; this time format is exclusively used thereafter and in the published dataset in REunion Time zone (RET) corresponding to UTC+4).

**Table 1:** The measured physical quantities (available in dataset 1).

Variable	Description	Unit	Sensor *Manufacturer*
GTI	Global Tilted Irradiance	W.m^−2^	SPN1 *Delta-T Devices* (cf. Fig. 4.a)
DTI	Diffuse Tilted Irradiance	W.m^−2^	
TA	Ambient temperature	°C	Thermocouple type K *TC SA* (cf. Fig. 4.b)
TPV	Back surface temperature of photovoltaic panel	°C	Thermocouple type T *TC SA* (cf. Fig. 4.c)
• Dataset 2: “dt2_electrical_production_inverter_1_with_faults.csv” provides electrical data from the first inverter, i.e., the one on which the faults were caused. Variables of the second dataset are described in Table 2 and the labels of the Fault are explained in Table 3.

**Table 2:** Electrical production variables (available in dataset 2 and 3).

Variable	Description	Unit
Eg	Energy injected to the grid	kWh
Pg	Power injected to the grid	W
Ia	Current produced by the PV plant	A
Ig	Current injected to the grid	A
Va	PV plant voltage	V
Vg	Grid voltage	V
Fg	Grid frequency	Hz
Fault	Fault type (cf. Table 3)

**Table 3:** Faults types description (available in dataset 2).

Value	Description	Detail
0	No-fault condition	
1	Uniform shading	
2.1	Constant partial shading - entire module	1 shaded PV module
2.2		2 shaded PV modules
2.3		3 shaded PV modules
3.1	Constant partial shading - portion of a module	1/3 shaded module
3.2		2/3 shaded module
4	Intermittent partial shading - static
• Dataset 3: “dt3_electrical_production_inverter_2.csv” contains data from inverter 2, the control inverter. The photovoltaic panels linked to this inverter are functioning normally and no fault has been produced. Variables of this dataset are described in Table 2.

Note that, the datasets concerning the electrical data (dataset 2 and 3) are dissociated since they are collected from two separated inverters. Data are stored by a datalogger Energrid with a sampling frequency of 0.2 Hz starting from 2021 to 10–01 for a minimum period of 12 months.

**Fig. 1:**
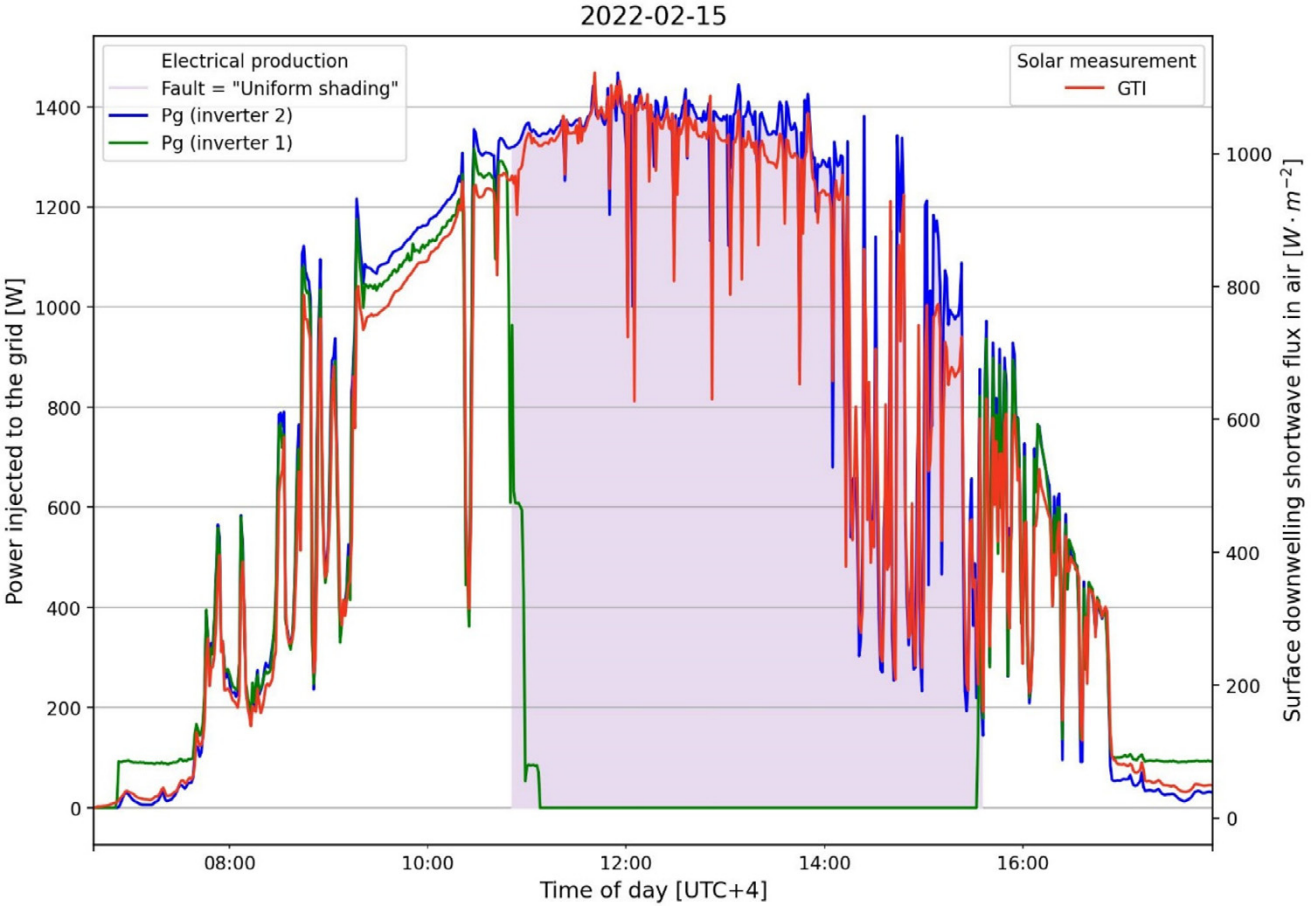
Measured irradiance (red, dataset 1), measured power of the control inverter (blue, dataset 3), measured power of the inverter connected to the PV line on which a uniform shading fault is caused (green, dataset 2). The purple zone depicts the fault period.

An example of 1-minute data is available for a single day as displayed in Fig. 1, including only limited variables: Global Tilted Irradiance (red), the two inverter AC powers output for the two photovoltaic panels lines (blue and green) when a uniform shading is generated (cf. Table 3: Fault=1).

## 3 Experimental Design, Materials and Methods

The studied system is a rooftop 4 kW PV plant, consisting of 2 lines of twelve polycrystalline modules, located on a building of University of La Reunion and oriented toward the north (the panels are oriented with an angle of 11° with the geographic north) with a tilt-angle of 26°

The first line includes TE1700 modules, and the second line TE1500 modules of Total Energie company. Each line consists of two parallel series of 6 panels. Each module contains 4 series of PV cells arranged horizontally (the 4 By-Pass diodes are on the right side of the module).

**Fig. 2:**
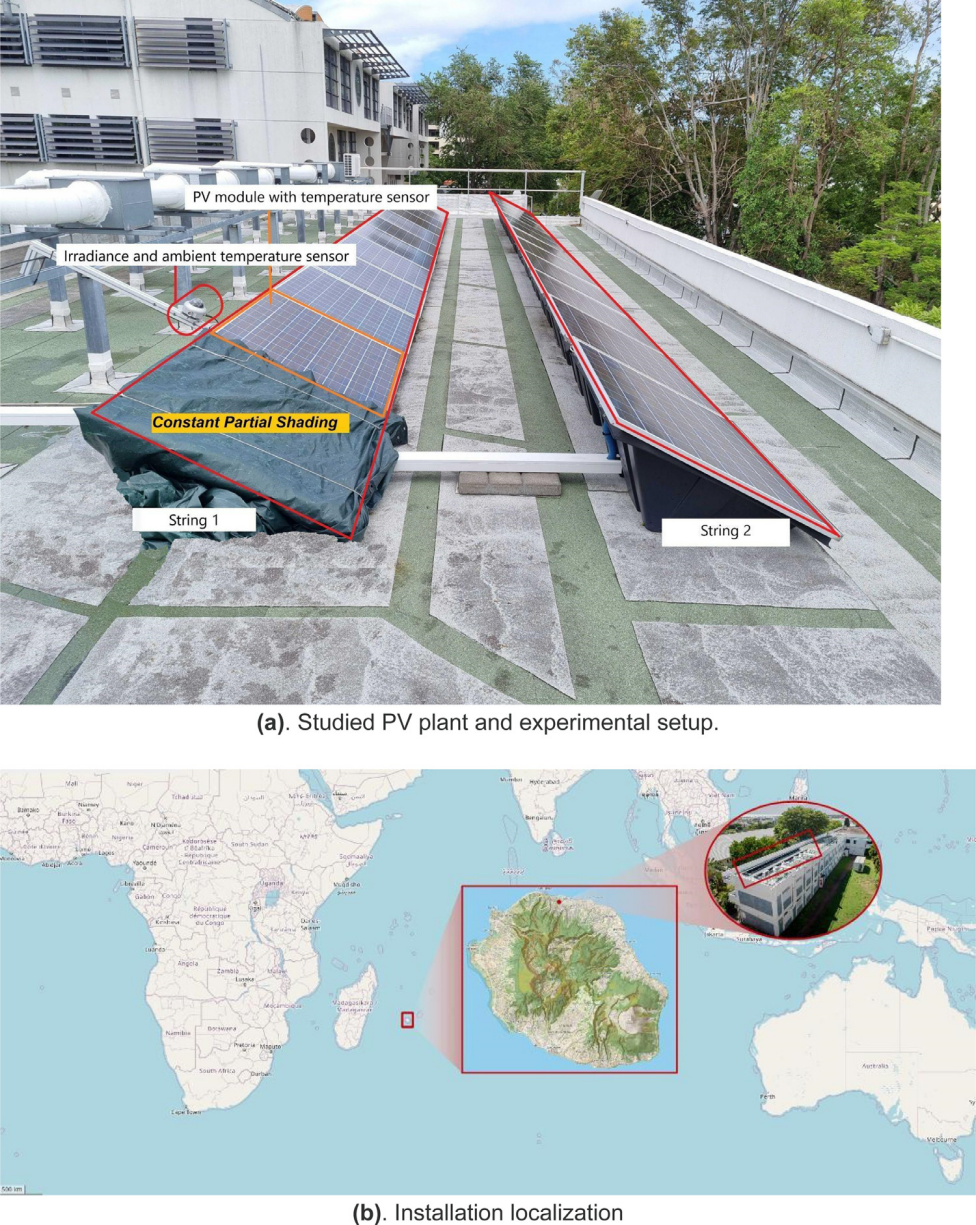
PV plant experimental setup and localization.

The overall installation can be seen in Fig. 2.a, and the installation world localization is exhibited in Fig. 2.b.

**Fig. 3:**
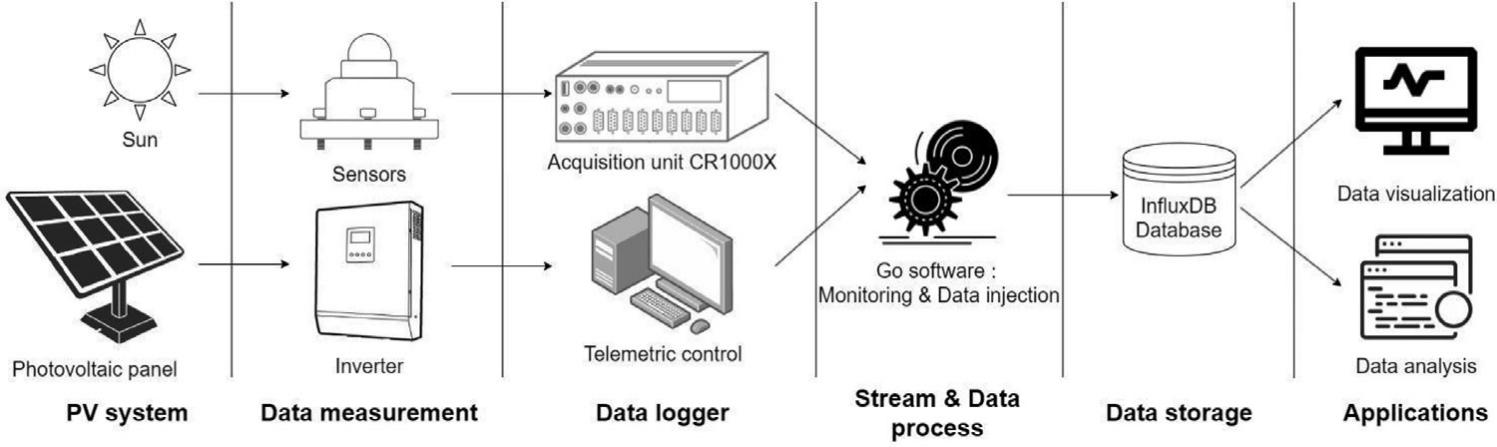
Diagram of the data pipeline.

Fig. 3 shows a diagram of the data acquisition process implemented to enable transformed and secure data transmission. This data pipeline consists of gateways, network services, a database to store the data, and application servers.

Electrical data measurements are acquired by an operating and management system for photovoltaic plants connected to the electrical network, coupled with inverters. The frequency of data repatriation has been selected from the telemetric control of the software.

**Fig. 4:**
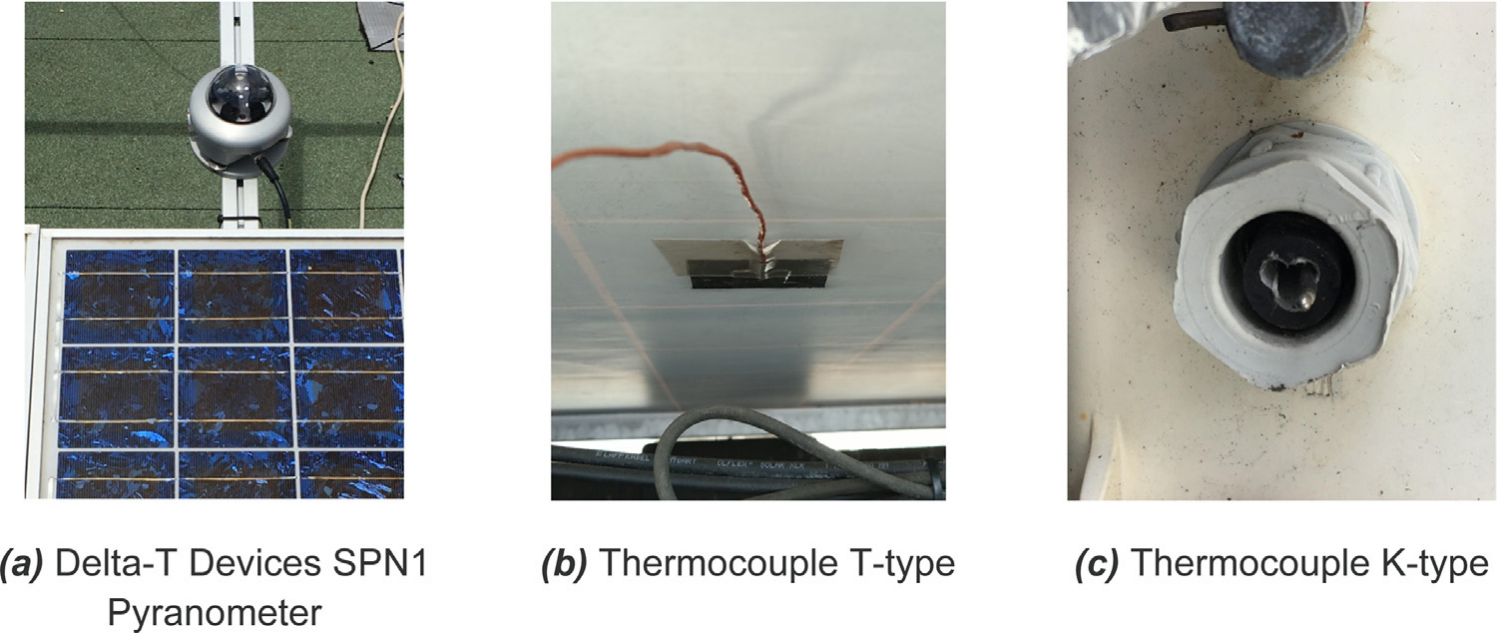
Solar and temperature sensors.

In order to monitor the solar radiation, ambient temperature and PV cell temperature, for the correlation of our studies, a pyranometer and two thermocouples have been installed. The Delta-T Devices SPN1 pyranometer, a precision solar radiation measurement instrument, has been installed to monitor the solar quantities needed to correlate our studies (Fig. 4.a). Ambient air temperature is acquired through a thermocouple type K TC-SA (Fig. 4.c). photovoltaic cell temperature is measured with a thermocouple type T TC-SA on the back surface of the module (Fig. 4.b), stuck under the module, in the middle of the second module of the line.

All radiometric and meteorological sensors are connected to a Campbell Scientific CR1000X data logger for data collection. The data logger is protected in a waterproof enclosure. The data is transmitted to the server via TCP/IP protocol. In order to evaluate the data in real time, the data is stored at a sampling rate of 1 Hz.

Sensors create a voluminous amount of data which places requirements for the servers, and clients too. As our devices broadcast their measurements in intervals of a couple of seconds, storing this data requires hard disk space, and processing larger time series datasets demands high computing power from clients. The solution uses InfluxDB 2.0 as a time-series database and the InfluxDB user interface for visualizations. The dashboard can be easily customized and is both locally and remotely accessible from any modern browser. Published studies [5] have shown that InfluxDB has advantages over many of the other options in performance, especially in executing aggregation functions over data and faster grouping of query results.

To ensure the availability of real-time data, software is written in the Go programming language. It monitors the data files from the various sources and injects data in the form of time series into the InfluxDB database. The program also manages the concatenation of the files locally and their organization through an established tree structure.

Accordingly, an end-to-end data pipeline that relies on open source technologies has been developed. Data from sensors and inverters are periodically sent, collected and stored in a centralized database system based on the Go program for real-time data streaming and InfluxDB for data storage. A set of customized dashboards has been created using the new features available since the last major InfluxDB release, to update real-time monitoring, data visualization and device status control.

**Fig. 5:**
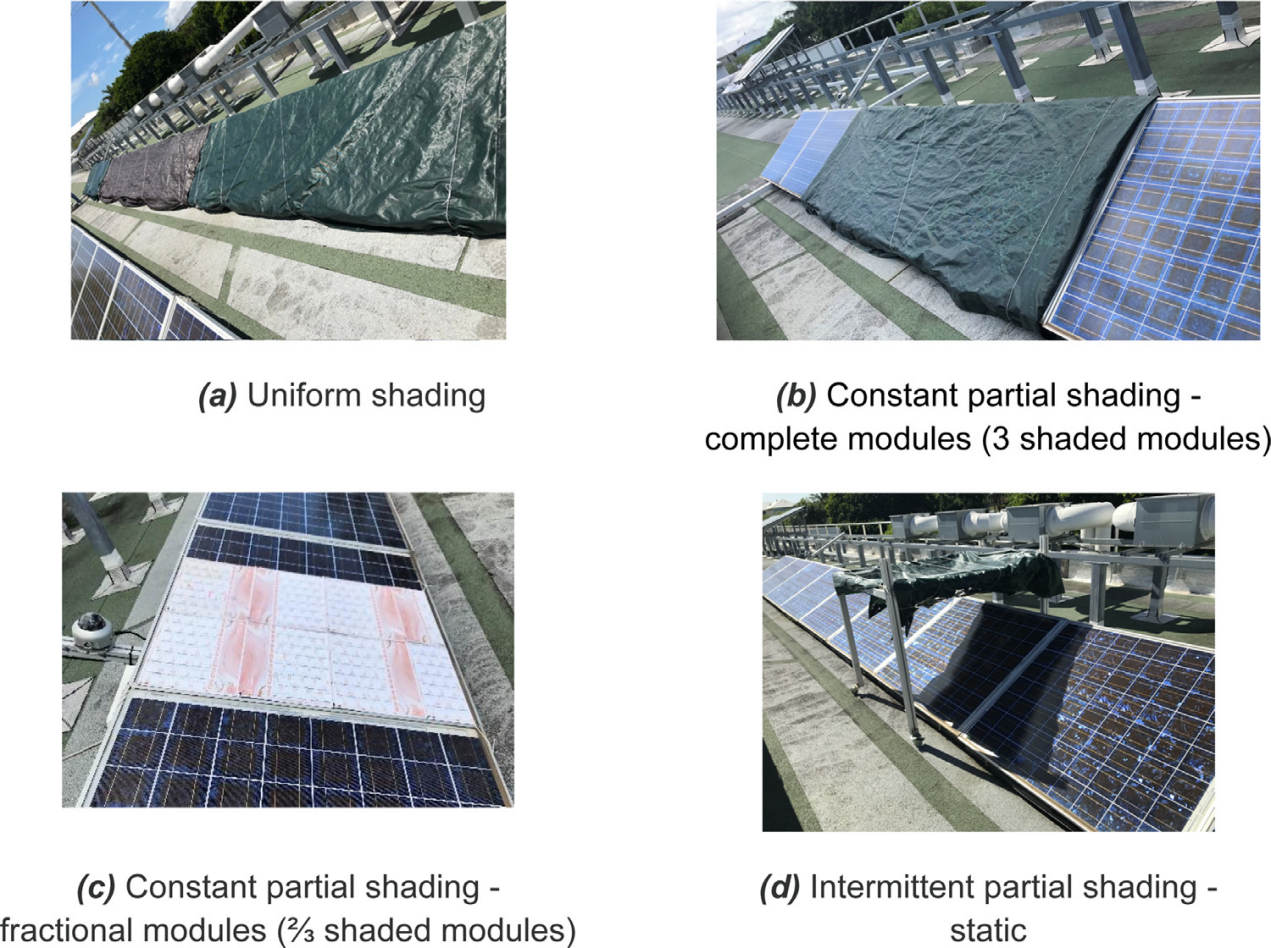
Shading experimental setup.

Shading faults are conducted thanks to different experimental setups during which the second line is kept in normal conditions. Fig. 5 exhibits the different experimental facilities depicted in Table 3 and are conducted as described below:

- Uniform shading: the entire line is obscured with an opaque material as shown in Fig. 5.a. Fault variable value is set at 1.
- Constant partial shading - entire module: 1, 2 or 3 modules are obscured with an opaque material (Fig. 5.b). Fault variable value is defined at 2.1, 2.2, and 2.3 respectively.
- Constant partial shading – portion of a module: an opaque mask is applied to one third (or two third) of the panel during a finite time in the daytime (Fig. 5.c). This configuration includes that each series of PV cells are impacted in different portions. Fault variable is set at the value of 3.1 and 3.2 respectively.
- Intermittent partial shading - static: An opaque object is fixed above the module line as exhibited in Fig. 5.d. The shadow of this object moves according to the path of the sun. This simulates the presence of a surrounding element shading the installation. Fault value is set at 4.

## Ethics Statements

There are no Ethics statements for the presented data.

## CRediT authorship contribution statement

**Alexandre Graillet:** Conceptualization, Methodology, Software, Validation, Investigation, Data curation, Writing – original draft. **Carole Lebreton:** Conceptualization, Methodology, Validation, Investigation, Writing – original draft. **Chao Tang:** Validation, Investigation, Writing – review & editing. **Fabrice Kbidi:** Conceptualization, Validation, Investigation, Resources, Data curation, Writing – review & editing, Project administration. **Tifenn Jegado:** Conceptualization, Methodology, Investigation, Validation. **Cédric Damour:** Resources, Supervision, Funding acquisition, Writing – review & editing. **Michel Benne:** Resources, Supervision, Funding acquisition.

## Declaration of Competing Interest

The authors declare that they have no known competing financial interests or personal relationships that could have appeared to influence the work reported in this paper.

## Data Availability

Electrical production data of a grid-connected rooftop PV plant in normal and shading faults conditions associated with solar and meteorological data in a tropical climate (Original data) (Zenodo)
